# LncRNA HOTAIR acts as competing endogenous RNA to control the expression of Notch3 via sponging miR-613 in pancreatic cancer

**DOI:** 10.18632/oncotarget.16462

**Published:** 2017-03-22

**Authors:** Huihua Cai, Jie Yao, Yong An, Xuemin Chen, Weibo Chen, Di Wu, Boyang Luo, Yong Yang, Yong Jiang, Donglin Sun, Xiaozhou He

**Affiliations:** ^1^ Department of Hepatobiliary Surgery, The First People's Hospital of Changzhou, The Third Hospital Affiliated to Soochow University, Changzhou, Jiangsu, China; ^2^ Department of Hepatobiliary and Pancreatic Surgery, Northern Jiangsu People's Hospital, The Clinic Medical College of Yangzhou University, Yangzhou, Jiangsu, China; ^3^ Department of Urology, The First People's Hospital of Changzhou, The Third Hospital Affiliated to Soochow University, Changzhou, Jiangsu, China

**Keywords:** pancreatic cancer, miR-613, HOTAIR, cell proliferation, invasion and migration

## Abstract

Pancreatic cancer is one of the most deadly cancers with a poor prognosis. Though studies have implicated the roles of microRNAs in pancreatic cancer progression, little is known about the role of miR-613 in pancreatic cancer. In the present study, the expression of miR-613 was down-regulated in pancreatic cancer tissues and cancer cell lines. Down-regulation of miR-613 was positively correlated with tumor differentiation, advanced TNM stage, nodal metastasis and shorter overall survival in patients with pancreatic cancer. Overexpression of miR-613 suppressed cell proliferation, invasion and migration, and induced cell apoptosis and cell cycle arrest at G0/G1 phase in pancreatic cancer cells. Bioinformatics analysis, luciferase reporter assay and rescue experiments showed that notch3 was a direct target of miR-613. MiR-613 was inversely correlated with notch3 expression in pancreatic cancer tissues. The long non-coding RNA, HOX transcript antisense RNA (HOTAIR) was up-regulated in both pancreatic cancer tissues and cancer cell lines, and HOTAIR suppressed the expression of miR-613 via functioning as a competing endogenous RNA. *In vivo* studies showed that stable overexpression of miR-613 or knock-down of HOTAIR suppressed tumor growth and also reduced the expression of notch3. In conclusion, these results suggest that HOTAIR functions as a competing endogenous RNA to regulate notch3 expression via sponging miR-613 in pancreatic cancer.

## INTRODUCTION

Pancreatic cancer is one of the most deadly cancers with a poor prognosis, and the overall 5-year survival rate is less than 5% due to advanced stage disease at initial diagnosis, frequent recurrence and the lack of effective therapies [1]. Surgical resection and chemotherapy were considered to be the main treatment options for pancreatic cancer [2]. Unfortunately, even with the advancement in medicine, pancreatic cancer is still resistant to current treatment regimens [3]. In this regard, it is important for us to understand the molecular mechanisms underlying pancreatic cancer progression.

Recently, the non-protein-coding portion of the genome is becoming more and more important in the regulation of normal physiology and the pathogenesis of diseases including cancer [4–6]. MicroRNA (miRNA) is a class of short non-coding RNAs, and it complements with the 3′ untranslated region (3′UTR) of mRNA and regulates the expressions of specific genes [7]. In the cancer studies, miRNAs can function to be oncogenic or tumor-suppressive. For example, miR-182 promotes pancreatic cancer cell proliferation and migration by targeting beta-TrCP2 [8]; miR-940 promotes cell proliferation by targeting GSK3β and sFRP1 in human pancreatic carcinoma [9]; on the other hand, miR-34a inhibits pancreatic cancer progression via Snail1-mediated epithelial-mesenchymal transition and the notch signaling pathway [10]; miR-377 inhibits the proliferation of pancreatic cancer by targeting Pim-3 [11]. The long non-coding RNAs (lncRNAs) are transcripts longer than 200 nucleotides, and they can not code proteins [12]. LncRNA was found to play important roles in various types of cancer including breast cancer, liver cancer, pancreatic cancer, colorectal cancer, gastric cancer and so on [12]. Various molecular mechanisms of lncRNA underlying cancer development have been proposed [13]. One of important mechanisms is that the lncRNA acts as a miRNA sponge to regulate the miRNA expression, which in turn regulates the expression of specific genes targeted by miRNA. Previously, our study has shown that the lncRNA, HOX transcript antisense RNA (HOTAIR) regulates the expression of miR-663b via the histone modification, which results in the regulation of pancreatic cancer progression [14]. In addition, HOTAIR was found to control the expression of Rab22a by sponging miR-373 in ovarian cancer [15]; Liu et al., showed that HOTAIR functions as a competing endogenous RNA to regulated human epidermal growth factor receptor 2 (HER2) expression by sponging miR-331-3p in gastric cancer [16]. Whether HOTAIR also functions as a competing endogenous RNA to regulate pancreatic cancer progression is largely unknown.

In the present study, we found that miR-613 was a potential target of HOTAIR by using bioinformatics tool. We then explored the role of miR-613 in pancreatic cancer. *In vitro* mechanistic studies revealed the tumor suppressive role of miR-613 via targeting neurogenic locus notch homolog protein 3 (notch3) in pancreatic cancer, and further study showed that HOTAIR functions as a competing endogenous RNA to regulate notch3 expression by sponging miR-613. *In vivo* and clinical results further confirmed the roles of miR-613 in pancreatic cancer progression. Therefore, our results may provide new insights into understanding the molecular mechanisms of miR-613 in pancreatic cancer.

## RESULTS

### The down-regulation of miR-613 in the pancreatic cancer tissues and cells lines

**Figure 1:**
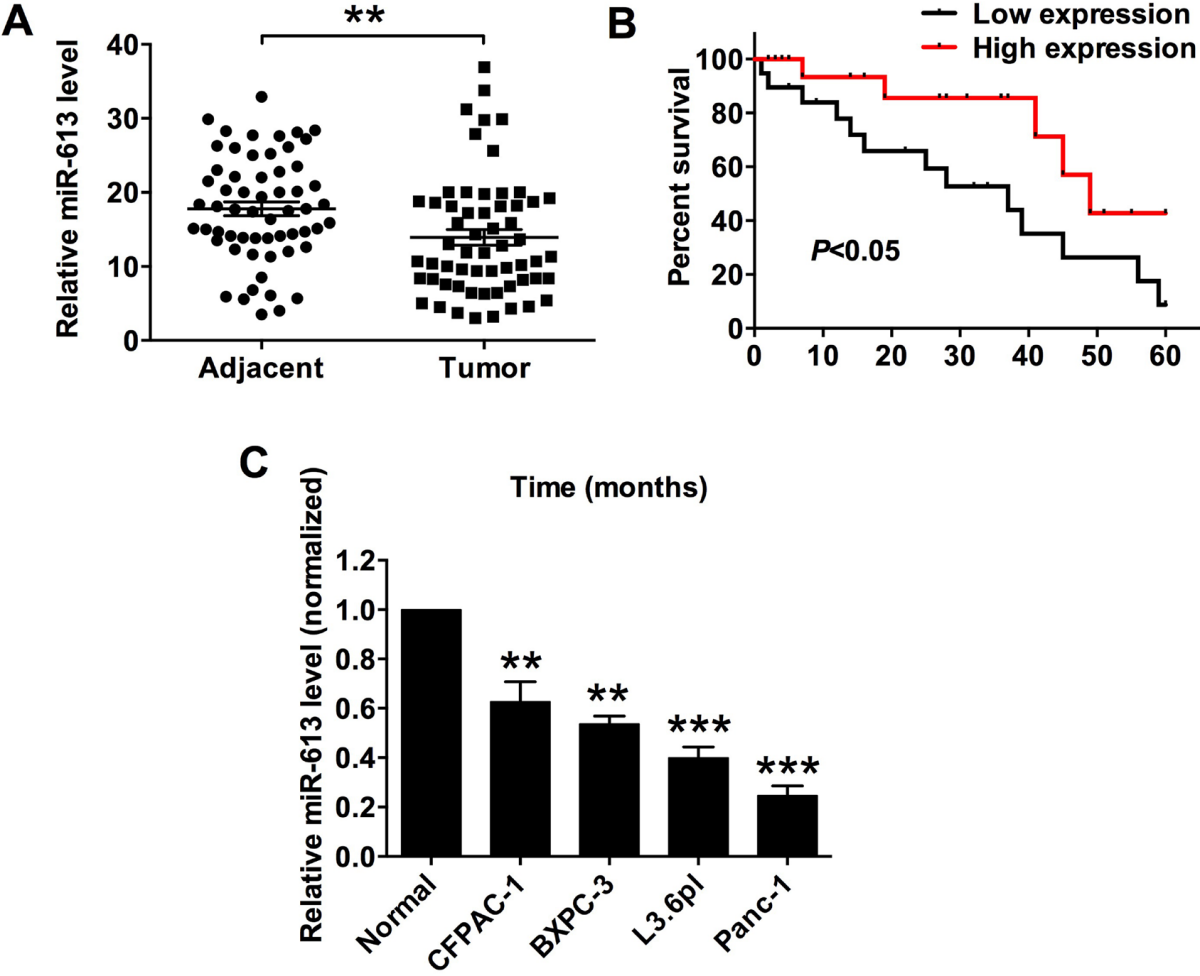
MiR-613 was down-regulated in pancreatic cancer tissues and cancer cells, and was correlated with poor survival in patients with pancreatic cancer patients (**A**) qRT-PCR analysis of miR-613 expression levels in adjacent normal pancreatic tissues and pancreatic cancer tissues from patients with pancreatic cancer. *N* = 59, significant difference between groups was shown as **P < 0.01 (Paired *t-test*). (**B**) Kplan-Meier survival analysis of patients with pancreatic cancer. Low expression level of miR-163 and high expression level of miR-613 was defined by using the median values of miR-613 expression levels in the 59 pancreatic cancer tissues. (**C**) qRT-PCR analysis of miR-613 expression levels in adjacent normal pancreatic cancer tissues and pancreatic cancer cell lines. *N* = 3, significant differences compared to adjacent normal pancreatic cancer tissues were shown as **P < 0.01, ***P < 0.001 (One-way ANOVA followed by Dunnett's test).

The miR-613 expression level in the clinical sample tissues from patients with pancreatic cancer was determined by the qRT-PCR assay, and the expression of miR-613 in the pancreatic cancer tissues was significantly lower than that in the adjacent normal pancreatic tissues (Figure 1A). The expression level of miR-613 in pancreatic cancer tissues was classified into low expression of miR-613 and high expression of miR-613 based on the median values of miR-613 expression in p. The association between miR-613 expression level and the clinicopathological parameters was analyzed in these 59 patients with pancreatic cancer, and low expression of miR-613 was significantly correlated with tumor differentiation, TNM stage and nodal metastasis, while miR-613 was not significantly associated with age, gender and tumor size (Table 1). We also followed the survival status of these patients, and the Kplan-Meier survival analysis showed that low expression of miR-613 was significantly correlated with shorter survival in patients with pancreatic cancer (Figure 1B) In addition, the expression levels in the pancreatic cancer cell lines including CFPAC-1, BXPC-3, L3.6pl and Panc-1 were also significantly down-regulated when compared to that in adjacent normal pancreatic tissues (Figure 1C).

**Table 1:** The association between miR-613 levels and clinicopathological characteristics of pancreatic cancer patients

	miR-613 expression		
	Low expression	High expression	*P*-value
	***N* = 28**	***N* = 31**	
Age			
< 56 years	12	17	0.4379
≥ 56 years	16	14	
Gender			
Male	15	12	0.3015
Female	13	19	
Tumor differentiation			
1–2	9	20	0.0191
3	19	11	
TNM stage			
I–II	7	17	0.033
III/IV	21	14	
Nodal metastasis			
0	7	19	0.0083
1	21	12	
Tumor size			
< 2 cm	10	18	0.1188
> 2 cm	18	13	

Low expression and high expression of miR-613 was determined by the cut-off values for miR-613, which were defined as the cohort median, and median of age was used as the cut-off values to define the subgroup (< 56 years old and ≥ 56 years old group). Statistical significance between groups was analyzed by Chi-square tests.

### Effect of miR-613 on pancreatic cell proliferation, cell invasion and migration, cell apoptosis and cell cycle

**Figure 2:**
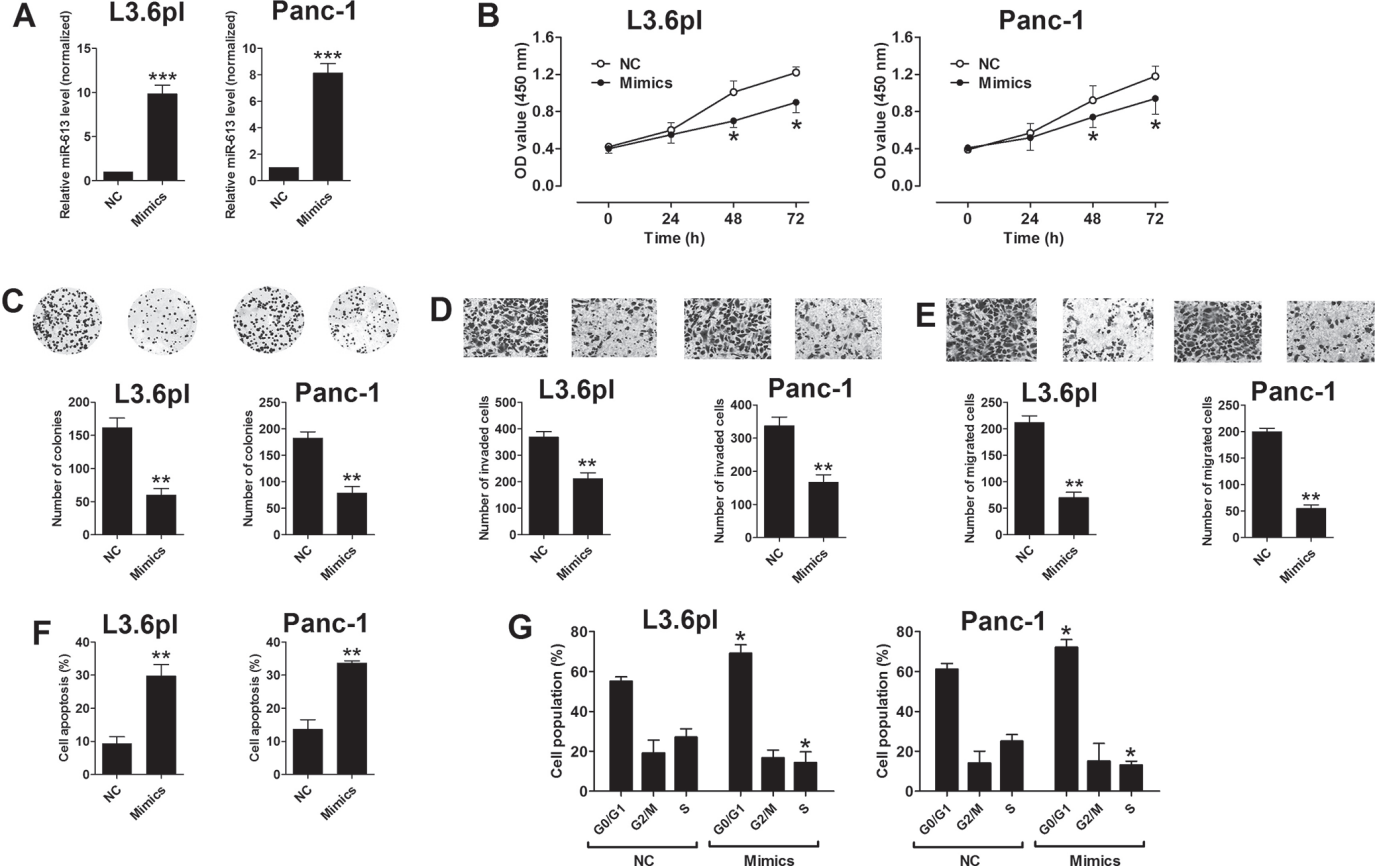
Up-regulation of miR-613 inhibited cell proliferation, invasion and migration in pancreatic cancer cells (**A**) qRT-PCR analysis of miR-613 expression levels in pancreatic cancer cells (L3.6pl and Panc-1) after miR-613 mimics or scrambled miRNA transfection. NC = negative control (scrambled miRNAs), mimics = miR-613 mimics, *n* = 3, significant differences between groups were shown as ***P < 0.001 (Unpaired *t-test*). (**B**) Cell proliferation of pancreatic cells (L3.6pl and Panc-1) after miR-613 mimics transfection or scrambled miRNA was determined by CCK-8 assay. *N* = 3, significant differences compared to NC group were shown as *P < 0.05 (Two-way ANOVA followed by Bonferroni's test). (**C**) Cell growth, (**D**) cell invasion, and (**E**) cell migration of pancreatic cells (L3.6pl and Panc-1) after miR-613 mimics or scrambled miRNA transfection were measured by colony formation assay, Transwell invasion assay, and Transwell migration assay, respectively. *N* = 3, significant differences compared to NC group were shown as **P < 0.01 (Unpaired *t-test*). (**F**) Cell apoptosis and (**G**) cell cycle of pancreatic cells (L3.6pl and Panc-1) after miR-613 mimics or scrambled miRNA transection was analyzed by flow cytometry. *N* = 3, significant differences compared to NC group were shown as *P < 0.05, **P < 0.01 (Unpaired *t-test*).

To further understand the molecular mechanisms of miR-613 in pancreatic cancer progression, we performed the gain-of-function studies. The overexpression of miR-613 in L3.6pl and Panc-1 cells was achieved by transient transfection with miR-613 mimics, and transfection with miR-613 mimics significantly increased the expression levels of miR-613 in L3.6pl and Panc-1 cells when compared to that transfected with scrambled miRNA (NC) (Figure 2A). The cell proliferative ability was measured by CCK-8 assay, and the cell proliferation was significantly suppressed in the pancreatic cells (L3.6pl and Panc-1) transfected with miR-613 mimics when compared to cells transfected with scrambled miRNA (Figure 2B). The cell growth was assessed by colony formation assay, and consistently, transfection with miR-613 mimics significantly suppressed the number of colonies formed by pancreatic cancer cells (L3.6pm and Panc-1) (Figure 2C). The cell invasion and migration were determined by Transwell invasion and Transwell migration assay, respectively, and the number of invaded and migrated cells in the miR-613 mimics-transfected group was significantly reduced when compared to the group transfected with scrambled miRNA (Figure 2D and 2E). The cell apoptosis and cell cycle were assessed by using flow cytometry experiments, and cell apoptosis was significantly increased by miR-613 transfection in the pancreatic cancer cells when compared to cells transfected with scrambled miRNA (Figure 2F). In the aspect of cell cycle, transfection with miR-613 mimics significantly increased the cell population in the G0/G1 phase accompanied by a decrease of cell population in the S phase in the pancreatic cancer cells (Figure 2G).

### MiR-613 repressed notch3 expression via targeting its 3′TUR

**Figure 3:**
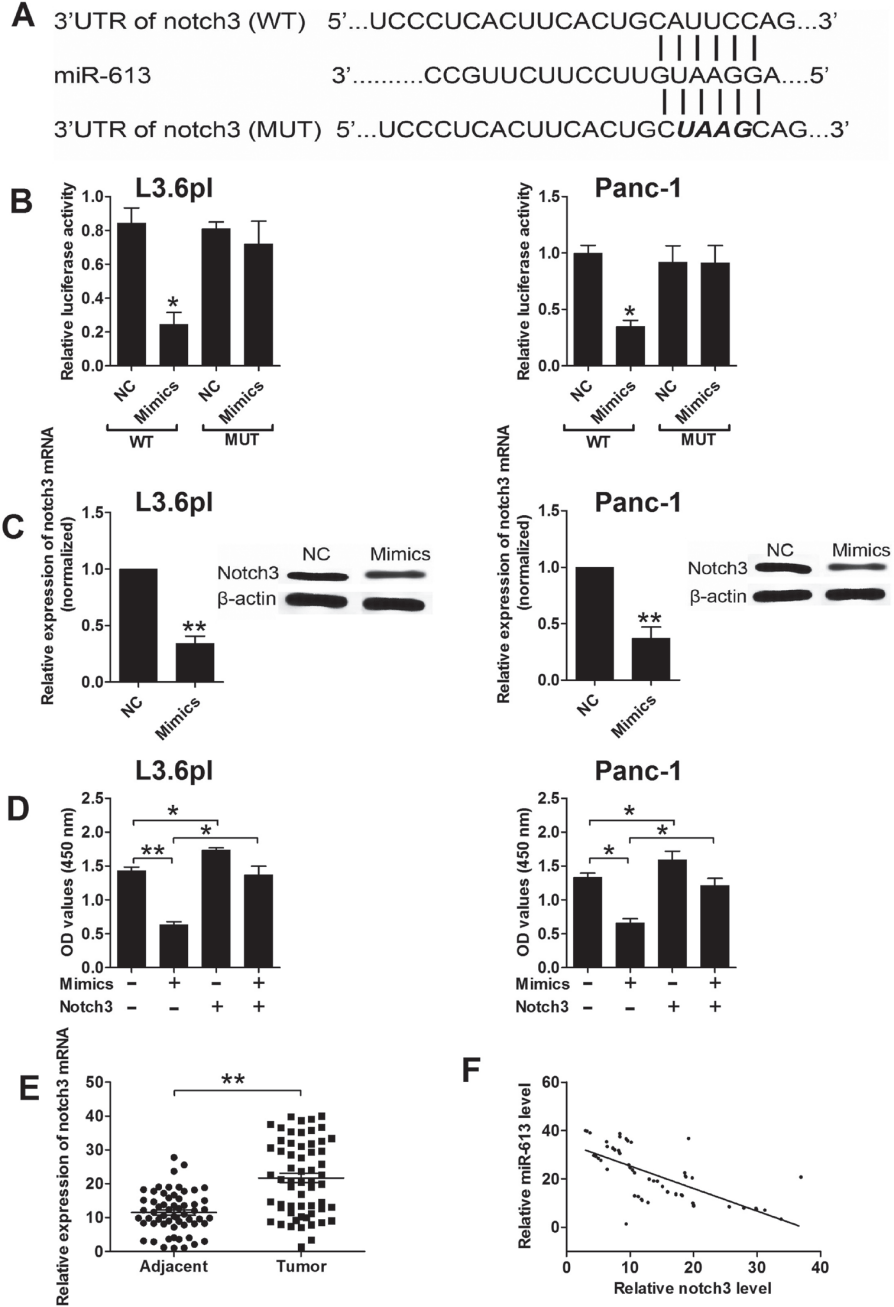
Notch3 was a downstream target of miR-613 in pancreatic cancer cells (**A**) Putative miR-613-binding sequences in the 3′UTR of notch3 and the reporter constructs showing the wild type (WT) notch3 3′UTR sequence and the mutated (MUT) notch3 3′UTR sequence. (**B**) miR-613 mimics suppressed the luciferase activity of the WT but not the MUT 3′UTR of notch3 reporter in pancreatic cells (L3.6pl and Panc-1). NC = negative control (scramble miRNAs), mimics = miR-613 mimics, *n* = 3, significant differences compared to NC group were shown as *P < 0.05 (Unpaired *t-test*). (**C**) qRT-PCR and western blotting analysis of notch3 mRNA and protein expression levels in pancreatic cancer cells (L3.6pl and Panc-1) after miR-613 mimics or scrambled miRNA transfection. *N* = 3, significant differences compared to NC group were shown as **P < 0.01 (Unpaired *t-test*). (**D**) Cell proliferation of pancreatic cancer cells (P3.6lp and Panc-1) co-transfected with miR-613 mimics (or scrambled miRNAs) and pcDNA3.1-notch3 (or pcDNA3.1 vector) was determined by CCK-8 assay. Mimics = miR-163 mimcis, nothc3 = pcDNA3.1-notch3, *n* = 3, significant differences among groups were shown as *P < 0.05, **P < 0.01 (One-way ANOVA followed by Dunnett's test). (**E**) qRT-PCR analysis of miR-613 levels in adjacent normal pancreatic tissues and pancreatic cancer tissues from patients with pancreatic cancer. *N* = 59, significant difference between groups was shown as **P < 0.01 (Paired *t-test*). (**F**) The inverse correlation between miR-613 and notch3 levels were analyzed by Pearson's correlation test (*R* = −0.6918, *P* < 0.001).

As miRNAs exert their function by targeting the 3′UTR of specific genes, we predicted the potential targets of miR-613 by using the TargetScan tool (www.targetscan.org), and predicted results showed that notch3 was one of the potential targets of miR-613. To confirm that notch3 was a target of miR-613, we performed the luciferase reporter assay. As shown in Figure 3A, we constructed the luciferase reporter plasmids containing the wide type 3′UTR of notch3 or the mutated 3′UTR of notch3, and pancreatic cancer cells transfected with miR-613 mimics and reporter plasmids containing the wide type 3′UTR of notch3 significantly suppressed the luciferase activity when compared to cells transfected with scrambled miRNA and reporter plasmids containing the wild type 3′UTR of notch3 (Figure 3B). Co-transfection with miR-613 mimics and mutated 3′UTR of notch3 had no effect on the luciferase activity when compared to cells co-transfected with scrambled miRNA and mutated 3′UTR of notch3 (Figure 3B). In addition, transfection with miR-613 mimics significantly suppressed the mRNA and protein expression levels of notch3 when compared to cells transfected with scrambled miRNA (Figure 3C). To further confirm the functional role in pancreatic cancer progression, cells co-transfected with scrambled miRNA and notch3 overexpressing plasmids (pcDNA3.1-notch3) significantly suppressed cell proliferation when compared to the control group, while co-transfection miR-613 mimics and notch3 overexpressing plasmids restored the inhibitory effects of miR-613 mimics in pancreatic cancer cells (Figure 3D). The notch3 mRNA expression level in the clinical sample tissues in these patients were also examined, and the mRNA expression level of notch3 in pancreatic cancer tissues was significantly up-regulated when compared to that in adjacent normal pancreatic tissues (Figure 3E). The Pearson's correlation analysis further showed that miR-613 expression level was inversely correlated with the expression level of notch3 mRNA in pancreatic cancer tissues (Figure 3F).

### Effects of HOTAIR on the miR-613 expression levels in pancreatic cancer cells

**Figure 4:**
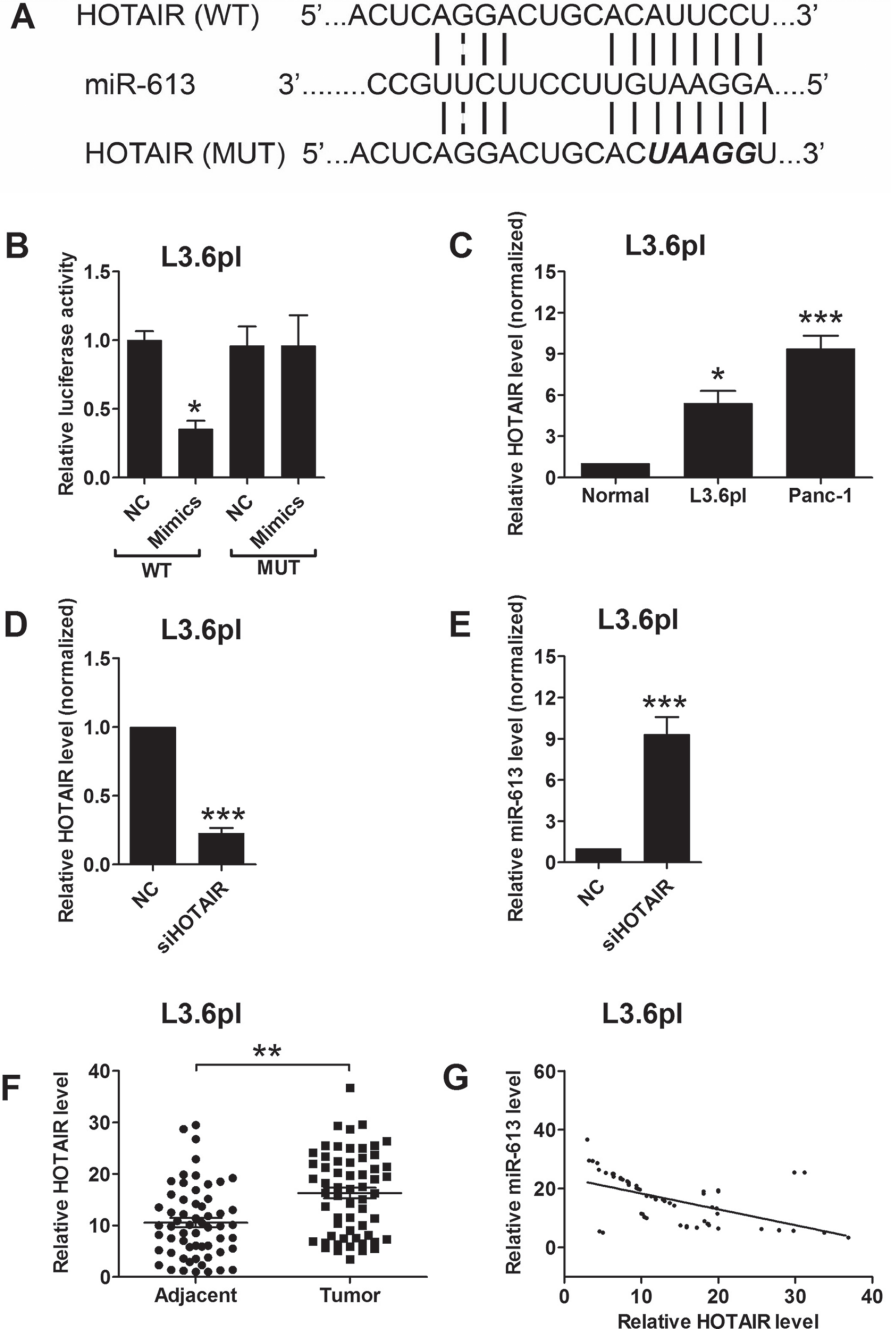
MiR-613 was inversely correlated with HOTAIR (**A**) Putative miR-613-binding sequences of HOTAIR and the reporter constructs showing the wild type (WT) HOTAIR sequence and the mutated (MUT) HOTAIR sequence. (**B**) miR-613 mimics suppressed the luciferase activity of the WT but not the MUT HOTAIR reporter in pancreatic cells (L3.6pl). *N* = 3, significant difference between groups was shown as *P < 0.05 (Unpaired *t-test*). (**C**) qRT-PCR analysis of miR-613 expression levels in adjacent normal pancreatic cancer tissues and pancreatic cancer cell lines. *N* = 3, significant differences compared to adjacent normal pancreatic cancer tissues were shown as *P < 0.05, ***P < 0.001 (One-way ANOVA followed by Dunnett's test). (**D**) qRT-PCR analysis of HOTAIR level in L3.6pl cells transfected with siHOTAIR or scrambled siRNA. *N* = 3, significant difference compared to NC group was shown as ***P < 0.001 (Unpaired *t* test). (**E**) qRT-PCR analysis of miR-613 level in L3.6pl cells transfected with siHOTAIR or scrambled lcnRNA. *N* = 3, significant difference compared to NC group was shown as ***P < 0.001 (Unpaired *t* test). (**F**) qRT-PCR analysis of HOTAIR levels in adjacent normal pancreatic tissues and pancreatic cancer tissues from patients with pancreatic cancer. *N* = 59, significant difference between groups was shown as **P < 0.01 (Paired *t-test*). (**G**) The inverse correlation between HOTAIR and miR663b levels were analyzed by Pearson's correlation test (*R* = −0.5455, *P* < 0.001).

HOTAIR was found to play an important role in pancreatic cancer progression, which has been shown in our previous study [14], and we further examined whether miR-613 had an interaction with HOTAIR. The bioinformatics prediction using the starBase tool [17, 18] found that HOTAIR was also a target of miR-613, and HOTAIR may act as a sponge for miR-613. To confirm the interaction between miR-613 and HOTAIR, we performed the luciferase reporter assay. As shown in Figure 4A, we constructed the luciferase reporter plasmids containing the wide type HOTAIR or the mutated HOTAIR (Figure 4A), and pancreatic cancer cells transfected with miR-613 mimics and reporter plasmids containing the wide type HOTAIR significantly suppressed the luciferase activity when compared to cells transfected with scrambled miRNA and reporter plasmids containing the wild type HOTAIR in L3.6pl cells (Figure 4B). Co-transfection with miR-613 mimics and mutated HOTAIR had no effect on the luciferase activity when compare to cells co-transfected with scrambled miRNA and mutated HOTAIR L3.6pl (Figure 4B). In agreement with previous study, HOTAIR was up-regulated in pancreatic cancer cells when compared to that in adjacent normal pancreatic tissues (Figure 4C). Transfection with HOTAIR siRNA markedly suppressed the expression level of HOTAIR (Figure 4D), and also increased the expression level of miR-613 in pancreatic cancer cells when compared to cells transfected with scrambled miRNA (Figure 4E). The expression level of HOTAIR was further confirmed to be up-regulated in the pancreatic cancer tissues when compared to that in adjacent normal pancreatic tissues (Figure 4F). More importantly, the expression level of miR-613 was inversely correlated with the expression level of HOTAIR in the pancreatic cancer tissues (Figure 4G).

### The effect of miR-613 and HOTAIR on the xenograft tumor growth *in vivo*

**Figure 5:**
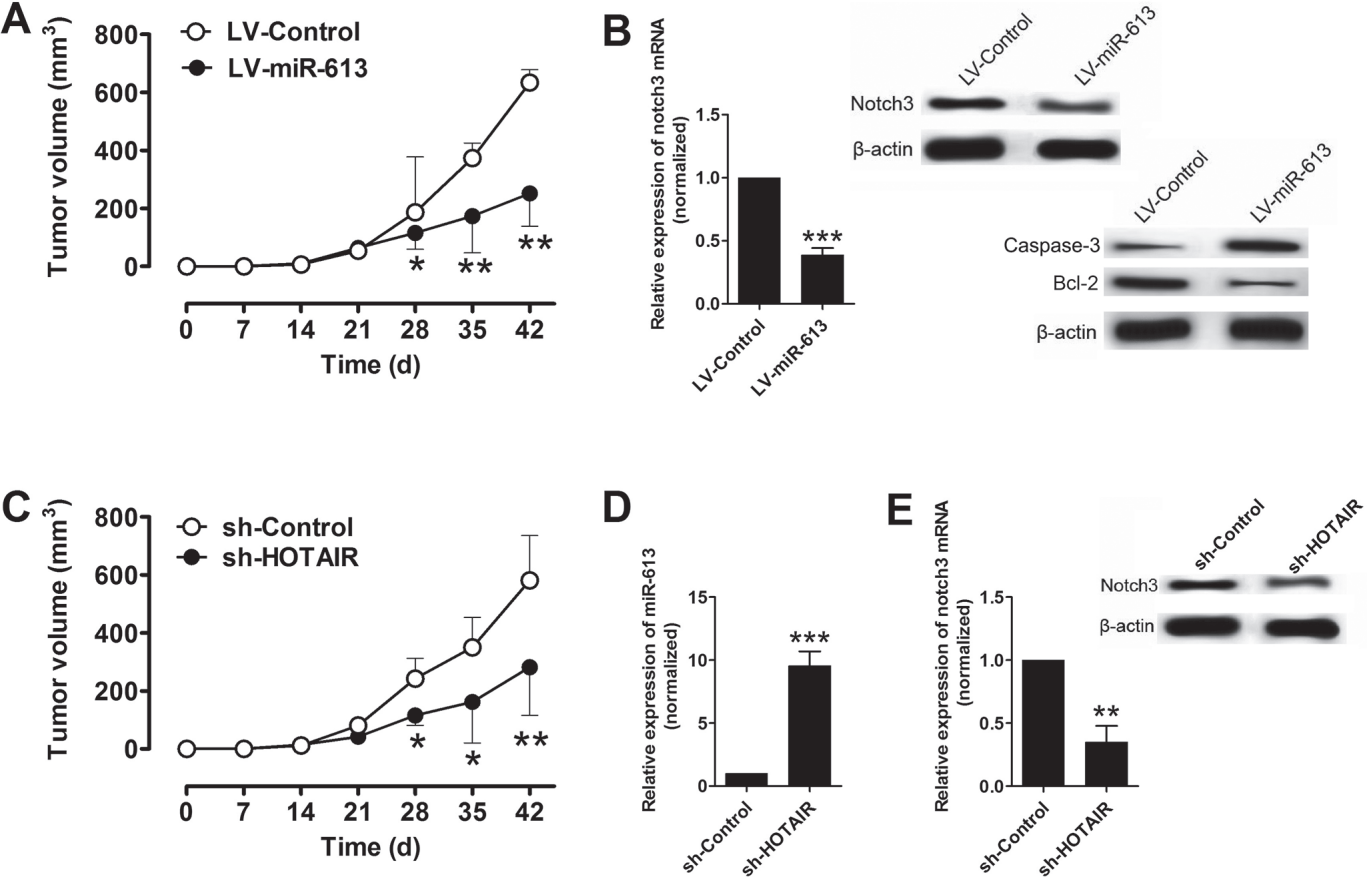
Overexpression of miR-613 or knockdown of HOTAIR suppressed xenograft tumor growth *in vivo* (**A**) Tumor volume changes in the mice bearing L3.6pl cells with miR-613 or scrambled miRNAs. *N* = 6, significant differences between groups were shown as *P < 0.05, **P < 0.01 (Two-way ANOVA followed by Bonferroni's test). (**B**) qRT-PCR and western blotting analysis of notch3 mRNA and notch3, caspase-3 and Bcl-2 protein levels in the tumor tissues isolated from the mice bearing L36.pl cells with miR-613 or scrambled miRNAs. *N* = 6, significant difference between groups was shown as ***P < 0.05 (Unpaired t test). (**C**) Tumor volume changes in the mice bearing L3.6pl cells transfected with sh-HOTAIR or sh-Control. *N* = 6, significant differences between groups were shown as *P < 0.05, **P < 0.01 (Two-way ANOVA followed by Bonferroni's test). (**D**) qRT-PCR analysis of miR-613 levels in the tumor tissues isolated from the mice bearing L3.6pl cells with sh-HOTAIR or sh-Control. *N* = 6, significant difference between groups was shown as ***P < 0.001 (Unpaired *t* test). (**E**) qRT-PCR and western blotting analysis of notch3 mRNA and protein levels in the tumor tissues isolated from the mice bearing L3.6pl cells with sh-HOTAIR or sh-Control. *N* = 6, significant difference between groups was shown as **P < 0.01 (Unpaired *t* test).

The role of miR-613 in pancreatic cancer was further examined *in vivo* by using the xenograft nude mice model. The nude mice were inoculated with L3.6pl cells transfected with LV-miR-613 or LV-Control, and in the LV-miR-613 transfected group, the tumor volume was significantly lower in LV-miR-613 group at 28, 35, and 42 d post inoculation than that in LV-Control group (Figure 5A). The mRNA and protein expression level of notch3 in the excised rumors were assessed by qRT-PCR and western blotting, respectively, and the mRNA and protein expression level of notch3 in the LV-miR-613 group were significantly lower than that in LV-Control group (Figure 5B), in addition, the protein levels of factors mediated apoptosis were also examined by western blotting, and the protein levels of caspase-3 was up-regulated and Bcl-2 was down-regulated in LV-miR-613 group (Figure 5B). We also inoculated the L3.6pl cells transfected with sh-HOTAIR or sh-Control, in the sh-HOTAIR group, the tumor volume was significantly lower at 28, 35, and 42 d post inoculation than that in the sh-Control group. In addition, the expression level of miR-613 was up-regulated in the excised tumors in the sh-HOTAIR group when compared to that in sh-Control group (Figure 5D). In addition, the mRNA and protein expression levels of notch3 were significantly lower in the tumors of sh-HOTAIR group than that in sh-Control group (Figure 5E).

## DISCUSSION

In the present study, our results showed that the expression of miR-613 was down-regulated in pancreatic cancer tissues and cancer cell lines. Down-regulation of miR-613 was positively correlated with tumor differentiation, advanced TNM stage, nodal metastasis and shorter overall survival in patients with pancreatic cancer. Overexpression of miR-613 inhibited cell proliferation, invasion and migration in pancreatic cancer cells. In addition, overexpression of miR-613 also induced apoptosis and cell cycle arrest at G0/G1 phase in the pancreatic cancer cells. Bioinformatics analysis, luciferase reporter assay and rescue experiment showed that notch3 was a novel a target of miR-613. Further bioinformatics analysis showed that HOTAIR functioned as a competing endogenous RNA to suppress the miR-613 expression. The effects of miR-613 on pancreatic cancer progression were further confirmed in the *in vivo* studies and in clinical samples.

Numerous studies have shown the important roles of miRNAs in the regulation of cancer progression. In pancreatic cancer, numerous miRNAs have been identified as either oncogenic miRNAs such as miR-191, miR-182, miR-940 [8, 9, 19] or tumor-suppressive miRNAs such as miR-34a and miR-454 [10, 20]. The role of miR-613 in pancreatic cancer has not been investigated. Previous studies have shown the tumor-suppressive role of miR-613 in various types of cancer including colorectal cancer, osteosarcoma, non-small cell lung cancer, papillary thyroid carcinoma, breast cancer, ovarian cancer, prostate cancer and esophageal squamous cell carcinoma [21–28]. Among these cancers, miR-613 was found to down-regulated in tumor tissues, and overexpression of miR-613 suppressed cell proliferation and cell invasion and migration. Consistently, our results for the first time showed that miR-613 was down-regulated in pancreatic cancer tissues and cell lines, and overexpression of miR-613 suppressed cell proliferation, cell invasion and migration. Further mechanistic study demonstrated that miR-613 overexpression induced cell apoptosis and caused cell cycle arrest at G0/G1 phase. Taken together, our results suggest that miR-613 plays a tumor-suppressive role in pancreatic cancer.

By using bioinformatics analysis, notch3 was predicted to be a downstream target of miR-613. Luciferase reporter assay and rescue experiment confirmed that miR-613 targets 3′UTR of notch3 to suppress the expression of notch3 and inhibits pancreatic cancer proliferation. Notch 3 is a protein that in encoded by the *NOTCH3* gene in human and is one of the important mediators in the Notch signaling pathway, which has been shown to maintain a pool of pancreatic progenitor cells at the early stage of pancreatic development, and governs pancreatic ductal cell differentiation [29]. Notch3 has been shown to be overexpressed in several types of cancers, which makes it a promising therapeutic target for cancer treatment [30]. Previous studies showed that notch3 was significantly elevated in pancreatic cancer tissues compared to normal pancreatic tissues, and the elevation of notch3 was also associated with more advanced tumors and tumor metastasis [31, 32]. In addition, notch3 was significantly associated with reduced overall and disease-free survival in patients with pancreatic cancer [31], and high levels of notch3 was positively correlated with high invasion and overall survival rates in pancreatic ductal adenocarcinoma [32]. The sensitivity of tarextumab treatment in combination with gemcitabine in pancreatic tumors was associated with higher levels of notch3 expression [33]. Further study also showed the notch3 could enhance sensitivity to gemcitabine in pancreatic cancer via inactivation of PI3K/Akt-dependent pathway [34]. Consistently, our results also showed the up-regulation of notch3 in pancreatic cancer tissues compared to the adjacent normal pancreatic tissues, and overexpression of notch3 attenuated the inhibitory effect of miR-613 in pancreatic cancer cells, suggesting the oncogenic role of notch3 in the pancreatic cancer. As miR-613 may have other potential downstream targets, some of which have been demonstrated in other types of cancers. The present study limited the investigation into notch3, and further study may be performed to examine the role of other targets of miR-613 in pancreatic cancer.

Human HOTAIR, is a 2.2 kb lncRNA transcribed from the HOXC locus and has been shown to bind both to the PRC2 and the LSD1 complexes and aberrant expression of HOTAIR has been associated with tumorigenesis in various types of cancers including breast cancer, colorectal cancer, lung cancer, gastric cancer [35–38]. Our previous study showed that HOTAIR regulated the expression of miR-663b via histone modification in pancreatic cancer [14]. Recently, HOTAIR was proposed to function as a competing endogenous RNA by sponging miRNAs to regulate miRNAs levels. Zhang et al, demonstrated that HOTAIR controls the expression of Rab22a by sponging miR-373 in ovarian cancer [15]. HOTAIR modulated c-KIT expression through sponging miR-193a in acute myeloid leukemia [39]. In addition, HOTAIR functions as a competing endogenous RNA to regulate HER2 expression by sponging miR-31-3p in gastric cancer [16]. Consistently, our data showed that HOTAIR was found to be up-regulated in both pancreatic cancer tissues and cell lines, and HOTAIR was inversely correlated with miR-613 level in pancreatic cancer tissues. Bioinformatics analysis and luciferase reporter assay showed that HOTAIR suppressed the expression miR-613 via sponging miR-613 in the pancreatic cancer cells. Knock-down of HOTAIR in L3.6pl cells suppressed the *in vivo* tumor growth and also suppressed the expression levels of miR-613, suggesting that the *in vivo* tumor suppressive role miR-613 may be correlated with HOTAIR. In summary, our results suggest the interaction between HOTAIR and miR-613 via “competing endogenous RNA” mechanism may be important for the pancreatic cancer progression.

In conclusion, the present study identified the interaction between HOTAIR and miR613 in pancreatic cancer. The results suggested that HOTAIR positively regulated the notch3 expression via acting as a competing endogenous RNA for miR-613 binding. The HOTAIR-miR-613-notch3 axis may be a promising therapeutic target for pancreatic cancer.

## MATERIALS AND METHODS

### Cell culture

The human pancreatic cancer cell lines (BXPC3, CFPAC-1, Panc-1 and L3.6pl) and HEK-293T cells were purchased from the Cell Bank of the Chinese Academy of Sciences (Shanghai, China). Cells were cultured in Dulbecco's modified Eagle's medium (DMEM, Sigma, St Louis, USA) supplemented with 10% fetal bovine serum (HyClone, GE Healthcare Life Science, Logan, USA) and incubated in a humidified chamber supplemented with 5% CO_2_ at 37°C.

### Tissue samples

Pancreatic cancer tissues and their matched adjacent normal pancreatic cancer tissues (approximately 5 cm from cancerous tissues) were taken from 59 patients undergoing surgery for pancreatic cancer at the First People's Hospital of Changzhou. All cases were reviewed by pathologist and histologically confirmed as pancreatic cancer based on histopathological evaluation. The characteristics of the patients were shown in Table 1. All tissues were immediately snap-frozen in liquid nitrogen and stored at −80°C until further experimentation. After surgical resection treatments, patients were further followed up every 2–4 months. No local or systemic treatment was conducted in these patients before surgical operation. Informed consents were obtained from all patients, and the study was approved by the Research Ethics Committee of the First People's Hospital of Changzhou.

### Oligonucleotide transfection, plasmid construction and lentiviral infection

MiR-613 mimics and its negative control, scrambled miRNAs were purchased from Ribobio (Guangzhou, China). The notch3 mRNA sequences were synthesized and subcloned into the pCDNA3.1 vector, and the empty pcDNA3.1 vector served as negative control (Genepharma, Shanghai, China). For HOTAIR, the siRNA specially targeting HOTAIR or its non-target negative control siRNA was synthesized by Genepharma. For transfection, L3.6pl cells or Panc-1 cells were grown on six-well plates to 60% confluence, and miRNA, siRNA or plasmid transfection was performed by using Lipofectamine 2000 (Invitrogen) according to the manufacturer's protocol. For the rescue experiment, cells were co-transfected with miRNAs (miR-613 mimics or scrambled miRNA) and plasmids (pcDNA3.1-notch3 or pcNDA3.1). Total RNA and protein were extracted at 24 h post transfection and used for qRT-PCR and western blot analysis.

MiR-613-overexpressing lentiviral constructs were generated using synthetic oligonucleotides and the Lv-CMV-GPF vector (Genepharma), and sh-HOTAIR-overexpressing lentiviral constructs were generated by subcloning sh-HOTAIR into pGLV3/H1/GFP lentiviral frame plasmids (Genepharma); empty vectors were used as negative controls, respectively. All the constructed plasmids were confirmed by sequencing (Invitrogen, Carlsbad, USA). Lentivirus carrying miR-613 or HOTAIR was packaged in the HEK293T cells and collected from the supernatants following the manufacturer's protocol. Stable cell lines for xenograft study were established by infecting the lentivirus into L36.pl cells.

### RNA extraction and qRT-PCR analysis

Total RNA was extracted from cells or tissues using TRIzol reagent (Invitrogen) according to the manufacturer's instruction. Notch3 mRNA level and HOTAIR was quantified by qRT-PCR using a SYBR Premix ExTaq Reverse Transcription PCR kit (Takaka, Dalian, China) and GAPDH was used as an internal control for normalization. For miR-613 detection, the miR-613 level was quantified by qRT-PCR using TaqMan assay kits (Applied Biosystems, Forster City, USA) and U6 was used an internal control for normalization. The primers for qRT-PCR were shown in Supplementary Table 1. The reaction was performed by using an ABI PRISM 7500 Sequence Detection System (Applied Biosystems). The relative expression levels of notch3 mRNA, HOTAIR and miR-613 were calculated using 2^−∆∆Ct^ method.

### CCK-8 assay

Cell proliferation assay was performed with Cell Counting Kit-8 (Dojindo, Kumamoto, Japan) according to the manufacturer's instruction. Twenty-four hour after transfection, cells were seed in 96-well plates at 1 × 10^4^ cells per well. The proliferative ability of cells was determined at 0, 24, 48, and 72 h. For the rescue experiment, cells were co-transfected with miRNAs (miR-613 mimics or scramble miRNA) and plasmids (pcDNA3.1-notch3 or pcNDA3.1), and the proliferative ability of cells was determined at 48 h. CCK-8 (10 μl) was added to each well at different time points, and the plate was incubated for 2 h at room temperature. The absorbance was measured at 450 nm using a microplate spectrophotometer (Molecular Devices, Sunnyvale, USA).

### Colony formation assay

Twenty-four hours after transfection, cells were seeded for colony formation in 6-cm culture dishes at a density of 1000 cells per dish. After continuous culture for 14 days, cells were fixed in methanol for 10 min and stained with 0.1% crystal violet for 30 min. Visible colonies were manually counted.

### Transwell invasion and migration assay

Transwell assay was performed using a chamber of 6.5 mm in diameter and with an 8-mm pore size (Corning, Corning, USA). For the invasion assay, twenty-four hours after transfection, 5 × 10^4^ cells in DMEM supplemented with 0.1% FBS were seeded onto the Matrigel-coated membrane matrix of the upper chamber, and the lower chamber was filled with DMEM supplemented with 10% FBS. After 24 h incubation, cells invading the bottom of the membrane were stained with 0.1% crystal violet in 20% ethanol, and the number of invaded cells was counted by using a DM2500 bright field microscope (LEICA, Wetzlar, Germany). For the migration assay, twenty-four hours after transfection, 5 × 10^4^ cells in DMEM supplemented with 0.1% FBS were seeded in the chamber of 6.5 mm of the upper chamber in diameter and with an 8-mm pore size (Corning), and the lower chamber was filled with DMEM supplemented with 10% FBS. After 24 h incubation, cells invading the bottom of the membrane were stained with 0.1% crystal violet in 20% ethanol, and the number of invaded cells was counted by using a DM2500 bright field microscope (LEICA).

### Cell apoptosis and cell cycle analysis

Twenty-four hour after transfection, cells were trypsinized and fixed with 70% ethanol for 30 min on ice. RNA was degraded by incubation with 20 mg/ml RNase (Sigma) for 1 h at 37°C. For the cell cycle analysis, cells were stained with propidium iodide at room temperature for 30 min, and then were analyzed by Calibur flow cytometry (BD Biosciences, Franklin Lakers, USA). For cell apoptosis analysis, cells were stained with FITC-Annexin V and propidum iodide (Beyotime, Beijing, China) and then were analyzed by Calibur flow cytometry (BD Biosciences) equipped with CellQuest software (BD Biosciences).

### Luciferase reporter assay

Cells were seeded in 96-well plates at 1 × 10^4^ cells per well. When the cells reached 60% confluence, they were co-transfected with wide type pGL3-notch3 3′UTR, mutated pGL3-notch3 3′UTR plasmids, wild type pGL3-HOTAIR, or mutated pGL3-HOTAIR and either scramble miRNA or miR-613 mimics using Lipofectamine 2000 (Invitrogen). Forty-eight hours after transfection, luciferase activity was measured with the Dual-Luciferase Reporter Assay System (Promega, Madison, USA) and expressed as the ratio between firefly and Renilla luciferase activities.

### Western blotting

Protein were extracted from cells or tissues using RIPA lysis buffer. Proteins were then separated by SDS-PAGE. After electrophoresis, proteins were transferred onto polyvinylidene difluoride membrane. After blocking with 5% non-fat milk for 2 h at room temperature, the membranes were then incubated with rabbit polyclonal notch3 antibody (ab23426; Abcam, Cambridge, UK), rabbit polyclonal caspase-3 (#9662; Cell Signaling Technology, Beverly, USA), mouse monoclonal Bcl-2 (#15071; Cell Signaling Technology) or mouse monoclonal β-actin (sc-47778; Santa Cruz) overnight at 4°C. The membranes were then incubated 2 h at room temperature with horseradish peroxidase-conjugated goat anti-rabbit or goat anti-mouse (Santa Cruz, Dalla, USA) secondary antibody and visualized with a chemiluminescence kit (Pierce, Rockford, USA).

### *In vivo* animal study

Tumor formation was studied by establishing a xenograft nude mice model. Four-week-old BALB/c nude mice were purchased from the Shanghai Experimental Animal Center (Chinese Academy of Sciences). The animal experiments in this study were approved by the Animal Research Committee of the First People's Hospital of Changzhou. Care and handling of the animals were in accordance with the guidelines for Institutional and Animal Care and Use Committees. A total of 24 animals were randomly divided into 4 groups with 6 animals in each group. Infected L3.6pl cells (1 × 10^6^/mice) were subcutaneously injected into the neck area of the nude mice. Tumor volumes (mm^3^) were measured every 7 days and calculated using the following formula: volume = width × length × height/2. The animals were killed 42 days after cell inoculation, and tumor tissues were harvested for further analysis.

### Statistical analysis

All the data were expressed as mean ± SD. Statistical analysis were performed by using Student's *t-test* or ANOVA followed by multiple comparison tests. The relationship between the expression of miR-613, notch3 and HOTAIR was examined by Pearson's correlation analysis. The correlation between miR-613 and pathological parameters was determined by Chi-square test. Differences were considered to be statistically significant when *P* < 0.05. All the results were performed in at least three independent experiments.

## SUPPLEMENTARY MATERIALS TABLES


