# Supplementary material for: LncRNA HOTAIR acts as competing endogenous RNA to control the expression of Notch3 via sponging miR-613 in pancreatic cancer
Source: Oncotarget. 2017 Mar 22;8(20):32905–17. doi: 10.18632/oncotarget.16462 (PMC5464837; doi:10.18632/oncotarget.16462)
Supplement: Supplementary file 1 [file oncotarget-08-32905-s001.pdf]

LncRNA HOTAIR acts as competing endogenous RNA to control the expression of Notch3 via sponging miR-613 in pancreatic cancer

Supplementary Materials

Supplementary Table 1: Primers for q-RTPCR experiment

	Forward primers	Reverse primers
Notch3	5′- TCTTGCTGCTGGTCATTCTC -3′	5′- TGCCTCATCCTCTTCAGTTG -3′
GAPDH	5′-AGAAAATCTGGCACCACACC-3′	5′-TAGCACAGCCTGGATAGCAA-3′
U6	5′-CTCGCTTCGGCAGCACA-3′	5′-AACGCTTCACGAATTTGCGT-3′
HOTAIR	5′-GGTAGAAAAAGCAACCACGAAGC-3′	5′-ACATAAACCTCTGTCTGTGAGTGCC-3′
